# Potential Adverse Drug Events and Drug–Drug Interactions with Medical and Consumer Cannabidiol (CBD) Use

**DOI:** 10.3390/jcm8070989

**Published:** 2019-07-08

**Authors:** Joshua D. Brown, Almut G. Winterstein

**Affiliations:** 1 Center for Drug Evaluation & Safety, Department of Pharmaceutical Outcomes & Policy, University of Florida College of Pharmacy, Gainesville, FL 32610, USA; 2 Department of Epidemiology, College of Public Health and Health Professions, University of Florida, Gainesville, FL 32610, USA

**Keywords:** cannabidiol, drug–drug interactions, adverse drug events, cannabis, safety

## Abstract

Cannabidiol (CBD) is ubiquitous in state-based medical cannabis programs and consumer products for complementary health or recreational use. CBD has intrinsic pharmacologic effects and associated adverse drug events (ADEs) along with the potential for pharmacokinetic and pharmacodynamic drug–drug interactions (DDIs). Given CBD use among patients with complex conditions and treatment regimens, as well as its expanded consumer use, awareness of potential safety issues with CBD is needed. Prescribing information for federally approved products containing CBD were reviewed. Data on ADEs and DDIs were extracted and summarized. Nearly one-half of CBD users experienced ADEs, which displayed a general dose-response relationship. Common ADEs include transaminase elevations, sedation, sleep disturbances, infection, and anemia. Given CBD effects on common biological targets implicated in drug metabolism (e.g., CYP3A4/2C19) and excretion (e.g., P-glycoprotein), the potential for DDIs with commonly used medication is high. General clinical recommendations of reducing substrate doses, monitoring for ADEs, and finding alternative therapy should be considered, especially in medically complex patients. CBD is implicated as both a victim and perpetrator of DDIs and has its own ADE profile. These effects should be considered in the risk-benefit assessment of CBD therapy and patients and consumers made aware of potential safety issues with CBD use.

## 1. Introduction

Cannabis (*Cannabis sativa* L.; or “marijuana”) is the most commonly used illicit substance worldwide [1]. The vast majority of cannabis use is recreational but there is increasing use of cannabis and cannabis-derived substances for medical and complementary health purposes. This increased access includes expansion of medical marijuana programs in roughly two-thirds of U.S. states and broad consumer marketing and use of cannabidiol (CBD) products [2]. These two sources of access can be thought to represent two distinct populations of users: (1) Those who are medically complicated and medically supervised with high comorbidity and pharmaceutical burden; and (2) the heterogeneous lay public using cannabis or derivatives for recreational or complementary health purposes without medical supervision, with or without other chronic health concerns and medications.

Both user populations, as well as much of the healthcare community, seemingly extrapolate the safety of cannabis and cannabis-derived substances like CBD from its long history of recreational use. For CBD specifically, safety is further assumed given CBD is currently broadly available and lacks the euphoric, psychoactive properties of tetrahydrocannabinol (THC). However, recognition that this general benignancy of CBD is perhaps only applicable to younger, healthier individuals using cannabis recreationally creates a tremendous patient safety concern in this new era. Particularly given the potential for other adverse drug events (ADEs) and drug–drug interactions (DDIs) with CBD.

CBD exhibits both pharmacodynamic (PD) and pharmacokinetic (PK) properties that could lead to ADEs and DDIs [3,4]. Whole cannabis and hemp (a strain grown to have low THC composition) contain over one hundred other cannabinoids and hundreds more botanical compounds that may have their own biological effects [5,6]. CBD may be delivered as a purified product, be one component in a botanical extract from cannabis or hemp, or be consumed as part of the whole cannabis or hemp plant. Regardless, CBD and other components in cannabis have known Cytochrome P450 (CYP450) and other enzyme system activity making these compounds susceptible to, or complicit as, inhibitors or inducers of these enzymes—i.e., PK-based DDIs [3]. Further, cannabis may have significant biological effects, e.g., sedation and somnolence, which can be potentiated with concomitant medications with similar effects (e.g., opioids or benzodiazepines)—i.e., PD-based DDIs [7,8]. Further, CBD and other cannabinoids have their own biological targets that may manifest as ADEs independent of any DDI potential.

## 2. Objectives

With the wide proliferation of CBD, the potential for public harm, and the dearth of evidence surrounding CBD’s potential for ADEs and DDIs, the objective of this narrative review was to summarize existing information regarding potential ADEs and DDIs with CBD from the prescribing information for approved pharmaceutical products and other existing literature for the use by clinicians and consumers. This review considers DDIs in the context of both medical and recreational/consumer use of CBD with consideration for CBD as both a victim and perpetrator of DDIs and synergistic ADEs. We further consider the underlying indications for CBD use and medications used for those disorders in the context of ADE/DDI risk and make general recommendations for co-prescribing (i.e., reduced dose) or avoiding certain combinations. We further introduce and discuss the regulatory environment for cannabis, hemp, and CBD as it pertains to the scope and magnitude of use.

## 3. Methods

For this review, full prescribing information or monographs and new drug applications (NDA) were extracted from federal agency websites (e.g., U.S. FDA, Health Canada). Products included two federally approved and regulated products containing CBD: Sativex (THC + CBD or “nabiximols”) [9] and Epidiolex (CBD) [10,11]. Other approved prescription products containing only derivatives of THC, dronabinol and nabilone, were out of scope. Prescribing information was reviewed and information on adverse events, clinical pharmacology, DDI studies, and contraindications were extracted and summarized. The review focused specifically on adverse reactions that could be attributable to DDIs or potentiated by concomitant use as well as ADEs that may be most relevant in medically complicated persons. Where necessary, human clinical trial publications were also reviewed. A focused literature review was also conducted to supplement information regarding PK/PD profiles of cannabis routes of administration, and prevalence of pharmacogenetic variants. DrugBank (https://www.drugbank.ca/) was used as a consistent drug information resource to describe potentially interacting, enzyme substrates, and pharmacodynamic effects throughout the review.

## 4. Results and Discussion

### 4.1. The Regulatory Environment of Medical Cannabis Use

CBD is increasingly used in state-approved medical cannabis programs. These programs range from what is deemed a “comprehensive” program that allows CBD and THC use (*N* = 23 states) or a restrictive program based on CBD with restrictions on the THC allowed (*N* = 13 states) while four states (ID, SD, NE, KS) have no program in place [2]. Currently, 11 states also have adult use cannabis programs (i.e., legalized recreational use). State-based medical cannabis programs were estimated to include over 2.1 million legal medical marijuana patients in 2018 with a range of ~1 to >38 users per 1000 state residents [12]. With much inter-state variation, practically all state programs have specified conditions for which medical cannabis can be legally used including, among others, epilepsy/seizures, chronic pain, nausea/vomiting, muscle spasms, inflammatory conditions (e.g., Cohn’s), Alzheimer’s and Parkinson’s disease, HIV/AIDS, and cancer [2]. Many of these conditions are highly prevalent in older adults, specific to children and adolescents with severe illness, or otherwise clustered among persons with serious underlying indications for medical cannabis use. Such users are likely to have complex medical profiles and pharmacotherapeutic regimens [13,14,15,16].

### 4.2. Increasing Consumer and Complementary CBD Use

CBD is expected to have potential for broad therapeutic use. Potential uses of CBD alone or in combination with THC include epilepsy, pain, cancer, inflammation, anxiety, neurodegeneration, multiple sclerosis, psychotic disorders, and depression [17,18,19,20,21,22,23]. Currently, only epilepsy, cancer-related pain, and multiple sclerosis are recognized indications for CBD by international federal agencies.

CBD-based consumer products have entered the U.S. market spurred in part by the 2018 “Farm Bill” [24], which effectively legalized hemp (with <0.3% THC) for agricultural purposes. CBD-infused products (including lip balms, beverages, vapors, edibles, topicals, essential oils, and so on) are highly prevalent in myriad consumer settings including gas stations, health spas, retail pharmacies, bakeries, and coffee shops. FDA has clearly stated that products that are specifically marketed with claims of a therapeutic benefit remain under the purview of the FDA and require approval – including cannabis- or hemp-derived CBD [25]. FDA further stated that no food products can enter inter-state commerce, CBD is not a safe food additive, and that CBD or other cannabis-derived compounds cannot be considered dietary supplements as they are or contain pharmacologically active ingredients [26]. Nevertheless, without clear enforcement, the products have proliferated primarily with CBD extracted from industrial hemp in concentrations ranging from very low (e.g., 20 mg soft drinks) to potentially super-therapeutic doses that exceed FDA-approved dosing for seizure disorders (e.g., gummies with 1500 mg CBD). By some estimates, the consumer CBD market alone may have reached sales of $600 million to $2 billion in 2018 and may approach $16 billion by 2025 [27]. Among people <35 years-old, more than 9% reported CBD use while 6.4% and 3.7% of those 45–55 years-old and 55+ years-old reported use at least once, but no information is available to assess the persistence for consumer use of CBD products [27].

### 4.3. Federally Approved CBD Products

Two CBD containing pharmaceutical products are currently marketed. These include Sativex^®^ (GW Pharmaceuticals) [9], a combined Δ-9-THC and CBD product indicated for spasticity and neuropathic pain in multiple sclerosis and as adjunctive analgesia for moderate to severe cancer pain. Sativex is approved in the United Kingdom, Europe, Canada, and other countries but has not been approved for use in the U.S. It is available as a buccal spray and is administered up to 12 sprays per day with 2.7 mg Δ-9-THC and 2.5 mg CBD per spray (30 mg max) [9].

Epidiolex contains only CBD in an oral solution with 100 mg/mL CBD. It is approved by the U.S. FDA for seizures associated with Lennox–Gastaut or Dravet syndromes only and is dosed by weight at a recommended 10 mg/kg daily (i.e., 500 mg for a 110-pound person) or a maximum of 20 mg/kg daily (1000 mg for a 110-pound person) [10]. Average doses in clinical trials slightly exceeded these approved doses and titration up to 50 mg/kg has been reported [28]. Table 1 summarizes key information regarding each product’s ingredients, dose, dosage form, and indications.

### 4.4. Potential for Adverse Drug Events and Drug–Drug Interactions

Potential for ADEs and DDIs is based on pharmacologic targets of CBD, pharmacodynamic effects, and interactions between CBD and other medications related to metabolism, absorption, and elimination. Descriptions of potential ADEs and DDIs is henceforth divided by description of the mechanism of the DDI or ADE, which included metabolic inhibition and induction; phase II metabolic pathways; drug transport; and pharmacodynamic effects. A brief discussion of CBD’s pharmacologic targets and effects is also included.

### 4.5. Molecular Targets of CBD

Cannabinoid (CB) receptors make up part of the endocannabinoid system, which leads to many of the therapeutic uses of cannabis product with roles in appetite, sleep, and pain sensations, as well as roles in the immune system, thermoregulation, and so on [29,30]. CB_1_ is the primary target for most desired therapeutic effects of THC but is also dose limiting given effects on mood, memory, and anxiety. CBD actually has low affinity for CB receptors and is considered a negative allosteric modulator of the endogenous cannabinoid, anandamide [4,31]. CBD may be an inverse agonist of CB_2_, which is strongly implicated in the immune system, and may contribute broad and varied anti-inflammatory effects [32,33,34]. Other than endocannabinoid dependent effects, CBD has several other recognized targets that may impart its therapeutic uses and harms including agonist activity at 5-HT1A/2A/3A serotonergic and TRPV-1 (vanilloid) receptors, antagonist activity on α-1 adrenergic and µ-opioid receptors, inhibition of synaptosomal uptake of noradrenaline, dopamine, serotonin, and gamma-amino butyric acid, inhibition of anandamide uptake, several ion channel effects, and activation of the peroxisome proliferator-activity receptor (PPAR)-γ.

A comprehensive review of these targets and potential effects can be found elsewhere [4]. Other molecular targets within the endocannabinoid system or independent pathways and their physiological actions are of continued interest [35,36]. Nevertheless, given these varied targets, CBD should be expected to have a variety of biological effects with documented large variation in dose requirements and response between individuals for both effectiveness and safety [35].

### 4.6. Metabolic Inhibition and Induction

CBD and its primary active metabolite 7-hydroxy CBD (7-OH-CBD) have similar reported effects on a number of CYP450 enzymes. CYP450 enzymes are implicated in the primary metabolism and biotransformation of the majority of therapeutic agents and xenobiotics [37]. Each product label reported, to varying degrees, CBD activity on one or more CYP450 isoforms including 3A4, 2C9, 2C19, 1A2, 2C8, 2B6, and 2E1 [9,10]. As a DDI “victim” the most relevant interactions were noted at 3A4 and 2C19 isoforms for CBD as these isoforms are responsible for the majority of biotransformation of CBD to the primary active metabolite (Table 2) [3]. As a DDI “perpetrator,” product labels suggest an inhibitory influence at clinically relevant concentration on 2C8, 2C9, 2D6, and 2C19 (Table 2). The label for Epidiolex also noted a possible dual inhibition/induction effect for 1A2 and 2B6.

There is little in vivo evidence related to CBD as a victim of metabolic DDIs. When co-administered with 3A4 inhibitors CBD and its active metabolite have increased systemic exposures and decreased exposures when co-administered with a 3A4 inducer. In a study where Sativex (four sprays) was co-administered with ketoconazole (400 mg; five days), a strong 3A4 inhibitor, CBD bioavailability increased by 89% [38]. In that scenario, 100% of the 36 healthy adult participants experienced an adverse event, primarily central nervous system in nature and possibly related to THC rather than CBD. When administered with rifampicin (600 mg; 10 days), a strong CYP3A4 and CYP2C19 inducer, CBD C_max_ decreased by 52%. Administration with omeprazole (40 mg; six days), a CYP2C19 inhibitor led to no changes [38].

One study tested in vivo DDI potential between CBD and co-administered clobazam, which is metabolized extensively by CYP3A4, CYP2C19, and CYP2B6, and also may be a competitive inhibitor on these isoforms [39]. Notably, CBD was observed to be a victim of this DDI with increases in C_max_ of 73% and AUC of 47% for CBD and 7-OH-CBD. Moreover, clobazam concentrations increased by a mean 60% and its active metabolite norclobazam was increased by 3–5-fold. These findings were confirmed in additional in vivo studies with additional observed increases in topiramate, rufinamide, zonisamide, and eslicarbazepine [40]. It is worth noting that these studies were prudent given the indication of Epidiolex for refractory and serious seizure disorders with concomitant antiepileptic therapies [11]. CDB has also been found to decrease in vitro activities of 3A5/7, 2D6, 2C9, 2A6, 2B6, 1A1, 1A2, 1B1, and 2J2 but the in vivo and clinical relevance of many of these interactions have not been established [3,4,41,42,43,44,45,46,47].

For the confirmed CYP450 isoforms 3A4 and 2C19 that are important to CBD metabolism, these enzymes are associated with some of the most common drugs implicated as inhibitors and inducers. These drugs have overlap with CBD and cannabis-related indications including epilepsy, chronic wasting disease in HIV/AIDS, and cancer. As CYP3A and CYP2C families are implicated in the metabolism of at least ~30% and ~25% of medications [37] the probability of these interactions is high. Thus, caution should be taken when using even medically supervised CBD in patients stabilized or newly initiating these medications in particular given the potential effects on both CBD and the medication in question. 

For recreational and consumer use, CYP3A4/2C19 inhibitors, substrates, and inducers are common medications representing common indications (e.g., hypertension, migraine, heartburn) used both acutely and chronically. These interactions can potentiate a wide array of ADEs and negative clinical outcomes specific to the substrate and indicated treatment. Thus, caution should be taken with any concomitant use between CBD and many common medications used by otherwise healthy persons. Further polymorphisms and actionable phenotypes of CYP2C19 and, to a lesser extent, CYP3A4, are not rare (~20%) and should be considered an additional source of variability and concern in the presence of CBD and other substrates of these enzymes [48,49]. Lastly, it should always be considered that CBD users might be exposed to one or more interacting drug, which can potentiate ADE/DDIs when multiple drugs overlap pharmacokinetically and/or pharmacodynamically.

### 4.7. Phase II Metabolic Pathways

Product labeling suggests CBD has inhibitory effects at clinically relevant dosing on UGT1A9, and UGT2B7 and recommends dosing changes in the presence of CBD (Table 3). Uridine 5’-diphospho-glucuronosyltransferase (UGT) enzymes catalyze glucuronidation of xenobiotics, a primary pathway of phase II metabolism, which creates a more easily excreted product; thus, inhibition of UGTs decreases excretion of the substrate. One particular in vitro study was cited using ethanol as a substrate and showed that CBD reduced UGT1A9 activity by 49% and UGT2B7 by 70% [50]. The clinical relevance of this activity has not been assessed. UGT1A9/2B7 substrates are summarized in Table 3 and are also among the most common medications used such as acetaminophen and ibuprofen and also common medications in complex patients who may use CBD such as tapentadol, canagliflozin, sorafenib, regorafenib, propofol, valproic acid, and mycophenolate. CBD should be used with caution in patients stabilized on or newly initiating these medications and side effects related specifically to the substrate’s toxicities should be monitored given UGT inhibition will decrease their excretion and increase bioavailability. Even basic, everyday over-the-counter medications are implicated, such as over-the-counter naproxen and ibuprofen could lead to significant side effects (e.g., bleeding) with both medical and consumer use of CBD.

### 4.8. Drug Transport, Absorption, and Efflux

CBD and its active 7-OH-CBD metabolite have no predicted activity on drug transporters. However, the inactive, hydroxylated, 7-COOH-CBD metabolite [10,51], which is also the most abundant metabolite, is a substrate for P-glycoprotein and an inhibitor of the breast cancer resistance protein (BCRP) and the bile salt export pump (BSEP) at clinically relevant concentrations. As the metabolite is inactive, no clinically relevant effects and actions are expected nor require CBD dose adjustments. However, both enzymes play roles in efflux of xenobiotics from tissues and transport into excretion pathways. For BCRP substrates, which include glyburide, imatinib, methotrexate, mitoxantrone, nitrofurantoin, prazosin, statins, dipyridamole, and others, increased side effects from these substrates are possible with increased distribution into tissues and decreased efflux into excretory organs. Similarly, BSEP substrates, such as paclitaxel, digoxin, statins, telmisartan, glyburide, ketoconazole, rosiglitazone, celecoxib, and others, can experience increased side effects. The general recommendations to avoid co-administration, monitoring for adverse effects and toxicity, and reducing the substrate dose when possible apply.

### 4.9. Synergistic Pharmacodynamic Effects

Extrapolations of common ADEs observed with CBD in clinical trials provide insight into synergistic DDIs that may occur. CBD is administered in patients with serious medical conditions that are treated with medications that have their own side effect profiles. Co-administration increases the potential of experiencing overlapping profiles even with direct DDIs via metabolic or transport pathways. CBD, compared to THC, does have noteworthy benefits in some complex patient profiles such as no addiction potential and fewer psychomimetic effects overall. However, these benefits and effects of THC are beyond the scope of this review.

While clinical trials of approved products provide insight to pharmacodynamic DDIs, caution should be taken in interpretation as the study cohorts include children and adults with serious epilepsy, multiple sclerosis, or other conditions and are not fully generalizable to all users. While we have reviewed both Sativex and Epidiolex prescribing information, this section includes a review mostly of Epidiolex prescribing information and other literature only as that product contains only CBD and many side effects associated with Sativex are indicative of THC rather than CBD such as psychoactive effects and cardiovascular warnings. Further, adverse effects of Epidiolex are reported for the recommended maintenance dose of 10 mg/kg/day as well as the maximum recommended dose of 20 mg/kg/day (Table 4). There is wide variation in consumer products with nearly half of CBD products reported to be underlabeled regarding CBD concentration and nearly one-third overlabeled as well as batch-to-batch and manufacturer variability [52], which may affect predictability of therapeutic response and ADEs. Prediction of risk-benefit should consider ADEs in light of expected dose variability, user weight, as well as other presented factors.

Further, variation will be introduced by “dosage” form, such as edible, vapor, or purified liquids as well as the dispensary or manufacturer [53]. Edibles have less predictable absorption profiles, which depends largely on the components of what is ingested (e.g., gummy bears vs. brownies) and is also representative of the erratic absorption that occurs when CBD is administered with food [54]. Liquids, especially when administered without food and at known concentrations, will have predictable bioavailability similar to Epidiolex and Sativex products with measurable drug levels between 1–3 h and max concentrations after 3–5 h after ingestion [54,55]. Vaping CBD, which is available via consumer products and some state-based CBD programs, will heat the mixture to approximately 180–200 Celsius and will lead to rapid bioavailability within the first inhalation with maximum concentrations reached within 15 to 30 min [56,57]. Vaping also risks transformation of CBD and any excipients to oxidized forms, though this is likely to a lesser extent compared to traditional smoking, as well as inter-user variability due to inhalation behaviors [58]. 

Considering variation in cumulative ADE risk, consumer use versus medical or complementary health use may vary. Many users may sporadically be exposed to CBD while others may consume it daily or multiple times per day. Inhibitory actions on metabolism or drug transport and pharmacodynamic interactions can be immediate in most cases whereas inductive effects require prolonged exposures (e.g., 21 days). Many ADEs such as somnolence, insomnia, and sleep disturbances are likely to occur even with sporadic and acute exposure while infections, transaminase elevations, and weight loss will require prolonged exposure.

Adverse effects associated with CBD appear to be dose dependent though not proportional in all cases. In phase III clinical trials, 2.7% versus 11.8% patients discontinued CBD treatment between the 10 mg/kg/day and 20 mg/kg/day treatment arms versus 1.3% for placebo.

#### 4.9.1. Transaminase Elevation and Hepatic Injury

The most frequent cause of discontinuation was transaminase elevation, which occurred in 8%, 16%, and 3% for the 10 mg/kg/day, 20 mg/kg/day, and placebo arms, respectively. Caution should be taken when CBD is used with medications with potential to cause hepatic injury or in people with pre-existing hepatic impairment, such as alcoholics or those with hepatitis. Such medications implicated with hepatic injury reports may include antiepileptics, antipsychotics, acetaminophen, certain antibiotics (e.g., amoxicillin and nitrofurantoin), antifungals, and verapamil. CBD as prescribed in Epidiolex carries a recommendation for a lowering by half for the starting, maintenance, and maximum doses (2.5, 5, and 10 mg/kg/day) with mild hepatic impairment (Child-Pugh A), reduction to 1.25, 2.5, and 5 mg/kg/day for moderate impairment (Child-Pugh B), and further reduction for severe (Child-Pugh C) of 0.5, 1, and 2 mg/kg/day [10].

#### 4.9.2. Somnolence, Sedation, and Asthenic Conditions

Common to cannabis-derived therapeutics are the general side effects of somnolence, sedation, lethargy, fatigue, and asthenia. In clinical trials, these occurred frequently in treatment groups and exhibited a modest dose-response relationship. Somnolence in particular occurred in 23% and 25% of patients treated with CBD (10 and 20 mg/kg/day), followed by fatigue (11% and 12%), lethargy (4% and 8%), and sedation (3% and 6%). Such side effects are also attributable to commonly prescribed medications such as benzodiazepines, opioids, antidepressants, antiepileptics, and antihistamines, which are used by medically complex and healthy persons alike. Co-administration will likely potentiate lethargic and sedative effects and may lead to excessive sedation, interruption in daily activities or work, and create a public health hazard via sedated drivers. Where possible, co-administration should be avoided, and patients and consumers counseled or informed of the potential for excessive sedation and steps to mitigate risk to themselves and others such as not operating vehicles or reserving CBD use for nighttime use. Clinical trials suggest that this side effect may also diminish with prolonged therapy so, when needed, a lower starting dose and slower titration may allow for continued CBD use until tolerance is achieved [11]. For recreational consumer use of CBD, such tolerance may never be achieved without sustained use.

#### 4.9.3. Insomnia and Sleep Disruption

Insomnia and sleep disruption were also observed with an inverse relationship to dose in 11% and 5% of patients in clinical trials. It is unclear if this dose-response relationship is related to target pathways and associated affinities for receptors or simply spurious. While a direct pathway for this adverse effect with CBD is uncertain, insomnia and sleep disruption are also side effects of other medications including antidepressants, dopamine agonists, stimulants, antiepileptics, steroids, diuretics, and beta-blockers. CBD users with these side effects should consider alternative regimens, dose of CBD or other medications, and the timing of doses. Additional pharmacotherapy to improve sleep with hypnotics or other sedatives would not be recommended given the aforementioned potential for excessive sedation. Sleep disturbances may also coincide with increased anxiety or mood changes, which should also not be managed by additional pharmacotherapy as the potential for ADE synergism is high.

#### 4.9.4. Suicidal Thoughts and Behavior

It is noted in prescribing information that all antiepileptic and many psychoactive medications have an increased risk of suicidal behavior and ideation, which is highest in epilepsy patients (3.5-fold increased relative risk). While not specifically assessed in a clinical study, prescribing information for Epidiolex mentions, though without any black box warnings, assessment of risk-benefit for CBD therapy related to suicide risk [10]. In addition, many common medications carry a similar risk as well as increase the overall risk of depression as a side effect. Antihypertensives, antidepressants, hormones, anxiolytics, analgesics, respiratory agents, and anticonvulsants all may have associated depressive or suicidal ideation or behaviors with an estimated prevalence of use of 20% and 10% for such medications in the general population. In addition to their common use, many of these medications have overlapping indications with CBD and, in general, depressive/suicidal behavior are higher in persons with serious chronic conditions [59,60]. Thus, the potential for such side effects is potentially high. As suggested before, CBD should be used in caution in persons using these other medications with a risk-benefit assessment before initiating treatment.

#### 4.9.5. Weight Loss, Infection, and Anemia

Other general side effects experienced by CBD users in clinical trials include weight loss, infections, and hematologic abnormalities. Weight loss is likely a result of decreased appetite, which was common with 16% and 22% of CBD-treated patients versus 5% in the placebo arm as well as increased diarrhea (9% and 20% vs. 9%). This can be complicated as other medications such as stimulants, antibiotics, chemotherapies, antiretrovirals, and some antidepressants also decrease appetite and increase weight loss. Particularly in cancer and HIV/AIDS, decreased appetite is a common indication for medical cannabis use and could conceivably be made worse with CBD in some users. While weight loss may be a desirable side effect for some users with other less serious conditions, weight loss or the underlying decreased appetite could complicate treatment, change how other medications are absorbed, or lead to other vitamin or mineral deficiencies. Complications can further include cardiovascular manifestations, liver damage, and osteoporosis if malnutrition is severe [61]. In such cases, users should consider supplementation where possible and, in more complex cases, discuss with doctors or pharmacists on the risk-benefit of using CBD in serious medical conditions.

Hematologic abnormalities were related to an increase in laboratory-defined anemia in 30% of treated patients versus 13% on placebo. The underlying mechanism is unknown; however, it may be due to decreased appetite and associated mineral deficiencies [61]. Common indications for CBD treatment, such as cancer, HIV/AIDS, Cohn’s, and other inflammatory disorders also increase the risk for anemia. Bone marrow suppression and subsequent anemia is also a side effect of chemotherapy, nonsteroidal anti-inflammatory drugs, and antibiotics [62,63], which may be more common in patients with complex healthcare needs. Further, some medications may decrease mineral and vitamin absorption, such as proton pump inhibitors and metformin, and lead to a worsening of this ADE if used concomitantly with CBD. Supplementation, when possible, should be considered when users experience anemic symptoms. 

Infection risk was 10% higher in CBD treated persons, particularly viral infections and pneumonia. Cannabinoids, via the CB-receptors, are thought to modulate the immune system and decrease immune response, particular of T-cell lymphocytes [4]. Caution should be considered in patients taking immunosuppressant medications (e.g., corticosteroids, tumor necrosis factor inhibitors) along with CBD, which will be common among persons using CBD for medical reasons. Along with a risk-benefit assessment of CBD therapy, users may consider vaccinations (e.g., pneumococcal and influenza) to increased immunity to such infections. 

For older patients with severe comorbidities in particular, it is noteworthy that Sativex carries a contraindication in its international product labeling for any users with pre-existing cardiovascular disease, which is likely attributable solely to THC sympathomimetic properties [4]. However, CBD has been observed to transiently reduce blood pressure in healthy adults [64]. In older adults, there may be potential to adversely lower blood pressure, in particular in individuals who are treated with antihypertensives (e.g., beta-blockers, alpha-agonists, diuretics) or other drugs that cause hypotension (e.g., nitrates, levodopa, tricyclic antidepressants), resulting in postural hypotension, syncope, falls, and possible fractures. Additional care should be taken in older persons taking medications with anticholinergic properties (e.g., antihistamines, etc.) and other sedating or psychoactive medications due to the possibility to increase risk of falls and injurious fractures and gait disturbances, all of which are also associated with CBD [11]. 

## 5. Conclusions

Contrary to popular belief and anecdotal evidence, CBD is not a biologically inert compound. Rather, CBD has a complex pharmacokinetic and pharmacodynamic profile similar to any other medication with the potential to interact with other medications and medical conditions. Medical CBD users under clinical supervision should be screened for potential DDIs and ADEs between CBD, other pharmacotherapies, and their underlying conditions. Increased awareness is needed among the lay public who are recreational or consumer CBD users. Healthcare providers should also be aware of the potential for DDIs and ADEs with CBD and strategically prescribe and manage patient regimens while also considering patient desires for complementary or alternative therapies.

## Figures and Tables

**Table 1:** Product information for cannabis-derived pharmaceutical products.

Product (Approval Date)	Active Ingredient(s)	Dosage Form	Route	Recommended Dose	Indication(s)
SATIVEX ^a^ (2011–2012)	Delta-9-THC and cannabidiol	Solution, spray	Buccal Spray	Titrated up to 12 sprays per day (patient median is 4–8 sprays). 2.7 mg THC and 2.5 mg CBD per spray.	Adjunctive treatment of spasticity and neuropathic pain in MS
					Adjunctive analgesic for moderate to severe pain in advanced cancer
EPIDIOLEX (2018) ^b^	Cannabidiol	Solution	Oral	2.5 mg/kg 2 × daily; maintenance 5 mg/kg 2 × daily; max 10 mg/kg 2 × daily	Seizures associated with Lennox–Gastaut or Dravet syndrome

^a^ Sativex is not approved in the United States but was approved in most other countries between 2011–2012. THC = tetrahydrocannabinol. ^b^ Cannabidiol doses up to 50 mg/kg have been reported in clinical trials.

**Table 2:** Metabolic drug–drug interactions between cannabidiol and enzyme substrates, inhibitors, or inducers.

Enzyme	Medication Examples	Effect/Recommendation
CYP3A4 substrates	Immunosuppressants, chemotherapeutics, antidepressants, antipsychotics, opioids, benzodiazepines, z-hypnotics, statins, calcium channel blockers, others	Increased risk of side effects related to substrate. Avoid co-administration, reduce substrate dose, monitor for adverse effects and toxicity. Avoid prescribing cascade with new treatment for side effects.
CYP3A4 inhibitors	Strong: Protease inhibitors, ketoconazole, loperamide, nefazodone Moderate: Amiodarone, verapamil, cimetidine, aprepitant, imatinib	Increased CBD bioavailability, possible increase in risk of adverse effects. Reduce CBD dose.
CYP3A4 inducers	Strong: Enzalutamide, phenytoin Moderate: Carbamazepine, topiramate, phenobarbital, rifampicin, efavirenz, pioglitazone	Decreased CBD bioavailability, possible decrease in CBD effectiveness. Increase CBD dose.
CYP2C19 substrates	Antidepressants, antiepileptics, proton pump inhibitors, clopidogrel, propranolol, carisoprodol, cyclophosphamide, warfarin	Increased risk of side effects related to substrate. Avoid co-administration, reduce substrate dose, monitor for adverse effects and toxicity. Avoid prescribing cascade with new treatment for side effects.
CYP2C19 inhibitors	Strong: Fluvoxamine, fluoxetine Other: Proton pump inhibitors, cimetidine, ketoconazole, clopidogrel, fluconazole, efavirenz	Increased CBD bioavailability, possible increase in risk of adverse effects. Reduce CBD dose.
CYP2C19 inducers	Rifampin, carbamazepine, phenobarbital, phenytoin, St. John’s Wort	Decreased CBD bioavailability, possible decrease in CBD effectiveness. Increase CBD dose.
CYP2C8/9 substrates	Rosiglitazone, burprenorphine, montelukast, celecoxib, sulfonylureas, losartan, naproxen, phenobarbital, phenytoin, rosuvastatin, valsartan, warfarin	Increased risk of side effects related to substrate. Avoid co-administration, reduce substrate dose, monitor for adverse effects and toxicity. Avoid prescribing cascade with new treatment for side effects.

**Table 3:** Drug–drug interactions between cannabidiol and secondary metabolism or transport proteins.

Enzyme	Medications	Effect/Recommendation.
UGT1A9	Regorafenib, acetaminophen, canagliflozin, sorafenib, irinotecan, propofol, mycophenolate, valproic acid, haloperidol, ibuprofen, dabigatran, dapagliflozin, others.	Increased risk of side effects related to substrate. Avoid co-administration, reduce substrate dose, monitor for adverse effects and toxicity.
UGT2B7	Hydromorphone, losartan, ibuprofen, naproxen, ezetimibe, lovastatin, simvastatin, carbamazepine, valproate, others.	
BCRP	Glyburide, imatinib, methotrexate, mitoxantrone, nitrofurantoin, prazosin, statins, dipyridamole	
BSEP	Paclitaxel, digoxin, statins, telmisartan, glyburide, ketoconazole, rosiglitazone, celecoxib	

UGT = uridine 5′-diphospho-glucoronosyltransferase; BCRP = breast cancer resistance protein; BSEP = bile salt export pump.

**Table 4:** Adverse events reported in clinical trials of cannabidiol (Epidiolex).

Adverse Events	Frequency ^a^		Other Medications with Similar ADE
	Cannabidiol	Placebo	
Transaminase elevation	8%, 16%	3%	Alcohol, acetaminophen, sulfonamides, antifungals, ACE inhibitors, antipsychotics
Somnolence, sedation, lethargy, fatigue	41%, 51%	15%	Benzodiazepines, opioids, antidepressants, antiepileptics, antihistamines
Decreased appetite	16%, 22%	5%	Stimulants, antibiotics, chemotherapies, antiretrovirals, some antidepressants
Diarrhea	9%, 20%	9%	Metformin, antibiotics, chemotherapy, proton pump inhibitors, antidepressants
Weight decreased	3%, 5%	1%	Stimulants, antibiotics, chemotherapies, antiretrovirals, some antidepressants
Insomnia, sleep disturbance	11%, 5%	4%	Antidepressants, dopamine agonists, stimulants, antiepileptics, steroids, diuretics, and beta-blockers
Gait disturbance	3%, 2%	<1%	Benzodiazepines, opioids, antidepressants, antiepileptics, antihistamines, antihypertensives, antiarrhythmics, sedatives/hypnotics, anticholinergics
Infections	41%, 40%	31%	Corticosteroids, tumor necrosis factor inhibitors, non-steroidal anti-inflammatory drugs, chemotherapy
Pneumonia	8%, 5%	1%	
Viral	7%, 11%	6%	
Suicidal thoughts or behaviors	Relative risk 1.8 to 3.5 ^b^	1.0	Antihypertensives, antidepressants, hormones, anxiolytics, analgesics, respiratory agents, and anticonvulsants

^a^ Reported respectively for Epidiolex doses of 10 mg/kg/day and 20 mg/kg/day. ^b^ Relative risk reported for all anti-epileptic drugs in a pooled meta-analysis. Highest (3.5) in patients with epilepsy indications.

## References

[1] Hall W., Renstrom M., Poznyak V. (2016). The Health and Social Effects of Nonmedical Cannabis Use.

[2] State Medical Marijuana Laws. http://www.ncsl.org/research/health/state-medical-marijuana-laws.aspx.

[3] Jiang R., Yamaori S., Takeda S., Yamamoto I., Watanabe K. (2011). Identification of cytochrome P450 enzymes responsible for metabolism of cannabidiol by human liver microsomes. Life Sci..

[4] Ibeas Bih C., Chen T., Nunn A.V., Bazelot M., Dallas M., Whalley B.J. (2015). Molecular Targets of Cannabidiol in Neurological Disorders. Neurotherapeutics.

[5] Elsohly M.A., Slade D. (2005). Chemical constituents of marijuana: The complex mixture of natural cannabinoids. Life Sci..

[6] ElSohly M.A., Radwan M.M., Gul W., Chandra S., Galal A. (2017). Phytochemistry of *Cannabis sativa* L.. Prog. Chem. Org. Nat. Prod..

[7] Bergamaschi M.M., Queiroz R.H., Zuardi A.W., Crippa J.A. (2011). Safety and side effects of cannabidiol, a *Cannabis sativa* constituent. Curr. Drug Saf..

[8] Iffland K., Grotenhermen F. (2017). An Update on Safety and Side Effects of Cannabidiol: A Review of Clinical Data and Relevant Animal Studies. Cannabis Cannabinoid Res..

[9] Sativex(R) (Delta-9-Tetrahydrocannabinol and Cannabidiol). https://www.bayer.ca/omr/online/sativex-pm-en.pdf.

[10] EPIDIOLEX (Cannabidiol) Prescribing Information. https://www.epidiolex.com/sites/default/files/EPIDIOLEX_Full_Prescribing_Information.pdf.

[11] Drug Approval Package: Epidiolex (Cannabidiol). https://www.accessdata.fda.gov/drugsatfda_docs/nda/2018/210365Orig1s000TOC.cfm.

[12] Number of Legal Medical Marijuana Patients. https://medicalmarijuana.procon.org/view.resource.php?resourceID=005889.

[13] Wong S.S., Wilens T.E. (2017). Medical Cannabinoids in Children and Adolescents: A Systematic Review. Pediatrics.

[14] Sexton M., Cuttler C., Finnell J.S., Mischley L.K. (2016). A Cross-Sectional Survey of Medical Cannabis Users: Patterns of Use and Perceived Efficacy. Cannabis Cannabinoid Res..

[15] Lucas P., Walsh Z. (2017). Medical cannabis access, use, and substitution for prescription opioids and other substances: A survey of authorized medical cannabis patients. Int. J. Drug Policy.

[16] Han B.H., Sherman S., Mauro P.M., Martins S.S., Rotenberg J., Palamar J.J. (2017). Demographic trends among older cannabis users in the United States, 2006–2013. Addiction.

[17] Whiting P.F., Wolff R.F., Deshpande S., Di Nisio M., Duffy S., Hernandez A.V., Keurentjes J.C., Lang S., Misso K., Ryder S. (2015). Cannabinoids for Medical Use: A Systematic Review and Meta-analysis. JAMA.

[18] Johnson J.R., Burnell-Nugent M., Lossignol D., Ganae-Motan E.D., Potts R., Fallon M.T. (2010). Multicenter, double-blind, randomized, placebo-controlled, parallel-group study of the efficacy, safety, and tolerability of THC:CBD extract and THC extract in patients with intractable cancer-related pain. J. Pain Symptom Manag..

[19] Da Rovare V.P., Magalhaes G.P.A., Jardini G.D.A., Beraldo M.L., Gameiro M.O., Agarwal A., Luvizutto G.J., Paula-Ramos L., Camargo S.E.A., de Oliveira L.D. (2017). Cannabinoids for spasticity due to multiple sclerosis or paraplegia: A systematic review and meta-analysis of randomized clinical trials. Complement. Ther. Med..

[20] Zuardi A.W., Shirakawa I., Finkelfarb E., Karniol I.G. (1982). Action of cannabidiol on the anxiety and other effects produced by delta 9-THC in normal subjects. Psychopharmacology.

[21] De Mello Schier A.R., de Oliveira Ribeiro N.P., Coutinho D.S., Machado S., Arias-Carrion O., Crippa J.A., Zuardi A.W., Nardi A.E., Silva A.C. (2014). Antidepressant-like and anxiolytic-like effects of cannabidiol: A chemical compound of Cannabis sativa. CNS Neurol Disord. Drug Targets.

[22] Leweke F.M., Piomelli D., Pahlisch F., Muhl D., Gerth C.W., Hoyer C., Klosterkotter J., Hellmich M., Koethe D. (2012). Cannabidiol enhances anandamide signaling and alleviates psychotic symptoms of schizophrenia. Transl. Psychiatry.

[23] Crippa J.A.S., Hallak J.E.C., Zuardi A.W., Guimaraes F.S., Tumas V., Dos Santos R.G. (2019). Is cannabidiol the ideal drug to treat non-motor Parkinson’s disease symptoms?. Eur. Arch. Psychiatry Clin. Neurosci..

[24] (2018). Agriculture Improvement Act of 2018.

[25] Gottlieb S. Statement from FDA Commissioner Scott Gottlieb, M.D., on Signing of the Agriculture Improvement Act and the Agency’s Regulation of Products Containing Cannabis and Cannabis-Derived Compounds. https://www.fda.gov/news-events/press-announcements/statement-fda-commissioner-scott-gottlieb-md-signing-agriculture-improvement-act-and-agencys.

[26] FDA Regulation of Cannabis and Cannabis-Derived Products: Questions and Answers. https://www.fda.gov/news-events/public-health-focus/fda-regulation-cannabis-and-cannabis-derived-products-questions-and-answers.

[27] Azer V., Blackledge J., Charles A.M., Chen O., Kernan J., Nadeua P., Neivert C., Osborne J., Rhyee C., Schenkel D. Cowen’s Collective View of CBD. Cowen’s Research. https://www.cowen.com/reports/cowen-collective-view-of-cbd/.

[28] Devinsky O., Marsh E., Friedman D., Thiele E., Laux L., Sullivan J., Miller I., Flamini R., Wilfong A., Filloux F. (2016). Cannabidiol in patients with treatment-resistant epilepsy: An open-label interventional trial. Lancet Neurol..

[29] Zou S., Kumar U. (2018). Cannabinoid Receptors and the Endocannabinoid System: Signaling and Function in the Central Nervous System. Int. J. Mol. Sci..

[30] Kaur R., Ambwani S.R., Singh S. (2016). Endocannabinoid System: A Multi-Facet Therapeutic Target. Curr. Clin. Pharmacol..

[31] Zhornitsky S., Potvin S. (2012). Cannabidiol in humans-the quest for therapeutic targets. Pharmaceuticals.

[32] Lunn C.A., Reich E.P., Bober L. (2006). Targeting the CB2 receptor for immune modulation. Expert Opin. Ther. Targets.

[33] Pertwee R.G. (2008). The diverse CB1 and CB2 receptor pharmacology of three plant cannabinoids: delta9-tetrahydrocannabinol, cannabidiol and delta9-tetrahydrocannabivarin. Br. J. Pharmacol..

[34] Pertwee R.G. (2005). Inverse agonism and neutral antagonism at cannabinoid CB1 receptors. Life Sci..

[35] Pisanti S., Malfitano A.M., Ciaglia E., Lamberti A., Ranieri R., Cuomo G., Abate M., Faggiana G., Proto M.C., Fiore D. (2017). Cannabidiol: State of the art and new challenges for therapeutic applications. Pharmacol. Ther..

[36] Brown A.J. (2007). Novel cannabinoid receptors. Br. J. Pharmacol..

[37] Zanger U.M., Schwab M. (2013). Cytochrome P450 enzymes in drug metabolism: Regulation of gene expression, enzyme activities, and impact of genetic variation. Pharmacol. Ther..

[38] Stott C., White L., Wright S., Wilbraham D., Guy G. (2013). A Phase I, open-label, randomized, crossover study in three parallel groups to evaluate the effect of Rifampicin, Ketoconazole, and Omeprazole on the pharmacokinetics of THC/CBD oromucosal spray in healthy volunteers. Springerplus.

[39] Geffrey A.L., Pollack S.F., Bruno P.L., Thiele E.A. (2015). Drug-drug interaction between clobazam and cannabidiol in children with refractory epilepsy. Epilepsia.

[40] Gaston T.E., Bebin E.M., Cutter G.R., Liu Y., Szaflarski J.P. (2017). Interactions between cannabidiol and commonly used antiepileptic drugs. Epilepsia.

[41] Jiang R., Yamaori S., Okamoto Y., Yamamoto I., Watanabe K. (2013). Cannabidiol is a potent inhibitor of the catalytic activity of cytochrome P450 2C19. Drug Metab. Pharmacokinet..

[42] Yamaori S., Okamoto Y., Yamamoto I., Watanabe K. (2011). Cannabidiol, a major phytocannabinoid, as a potent atypical inhibitor for CYP2D6. Drug Metab. Dispos..

[43] Yamaori S., Kushihara M., Yamamoto I., Watanabe K. (2010). Characterization of major phytocannabinoids, cannabidiol and cannabinol, as isoform-selective and potent inhibitors of human CYP1 enzymes. Biochem. Pharmacol..

[44] Yamaori S., Okushima Y., Yamamoto I., Watanabe K. (2014). Characterization of the structural determinants required for potent mechanism-based inhibition of human cytochrome P450 1A1 by cannabidiol. Chem. Biol. Interact..

[45] Yamaori S., Koeda K., Kushihara M., Hada Y., Yamamoto I., Watanabe K. (2012). Comparison in the in vitro inhibitory effects of major phytocannabinoids and polycyclic aromatic hydrocarbons contained in marijuana smoke on cytochrome P450 2C9 activity. Drug Metab. Pharmacokinet..

[46] Yamaori S., Ebisawa J., Okushima Y., Yamamoto I., Watanabe K. (2011). Potent inhibition of human cytochrome P450 3A isoforms by cannabidiol: Role of phenolic hydroxyl groups in the resorcinol moiety. Life Sci..

[47] Arnold W.R., Weigle A.T., Das A. (2018). Cross-talk of cannabinoid and endocannabinoid metabolism is mediated via human cardiac CYP2J2. J. Inorg. Biochem..

[48] Zhou Z.W., Chen X.W., Sneed K.B., Yang Y.X., Zhang X., He Z.X., Chow K., Yang T., Duan W., Zhou S.F. (2015). Clinical association between pharmacogenomics and adverse drug reactions. Drugs.

[49] Van Driest S.L., Shi Y., Bowton E.A., Schildcrout J.S., Peterson J.F., Pulley J., Denny J.C., Roden D.M. (2014). Clinically actionable genotypes among 10,000 patients with preemptive pharmacogenomic testing. Clin. Pharmacol. Ther..

[50] Al Saabi A., Allorge D., Sauvage F.L., Tournel G., Gaulier J.M., Marquet P., Picard N. (2013). Involvement of UDP-glucuronosyltransferases UGT1A9 and UGT2B7 in ethanol glucuronidation, and interactions with common drugs of abuse. Drug Metab. Dispos..

[51] Ujvary I., Hanus L. (2016). Human Metabolites of Cannabidiol: A Review on Their Formation, Biological Activity, and Relevance in Therapy. Cannabis Cannabinoid Res..

[52] Bonn-Miller M.O., Loflin M.J.E., Thomas B.F., Marcu J.P., Hyke T., Vandrey R. (2017). Labeling Accuracy of Cannabidiol Extracts Sold Online. JAMA.

[53] Madras B.K. (2017). Are THC Levels in Oral Fluids and Blood Plasma Comparable after Oral Ingestion of Edibles Containing Cannabis or THC?. Clin. Chem..

[54] Vandrey R., Herrmann E.S., Mitchell J.M., Bigelow G.E., Flegel R., LoDico C., Cone E.J. (2017). Pharmacokinetic Profile of Oral Cannabis in Humans: Blood and Oral Fluid Disposition and Relation to Pharmacodynamic Outcomes. J. Anal. Toxicol..

[55] Karschner E.L., Darwin W.D., Goodwin R.S., Wright S., Huestis M.A. (2011). Plasma cannabinoid pharmacokinetics following controlled oral delta9-tetrahydrocannabinol and oromucosal cannabis extract administration. Clin. Chem..

[56] Schwope D.M., Karschner E.L., Gorelick D.A., Huestis M.A. (2011). Identification of recent cannabis use: Whole-blood and plasma free and glucuronidated cannabinoid pharmacokinetics following controlled smoked cannabis administration. Clin. Chem..

[57] Abrams D.I., Vizoso H.P., Shade S.B., Jay C., Kelly M.E., Benowitz N.L. (2007). Vaporization as a smokeless cannabis delivery system: A pilot study. Clin. Pharmacol. Ther..

[58] Martin J.H., Schneider J., Lucas C.J., Galettis P. (2018). Exogenous Cannabinoid Efficacy: Merely a Pharmacokinetic Interaction?. Clin. Pharmacokinet..

[59] Ferro M.A. (2016). Major depressive disorder, suicidal behaviour, bipolar disorder, and generalised anxiety disorder among emerging adults with and without chronic health conditions. Epidemiol. Psychiatr. Sci..

[60] Fassberg M.M., Cheung G., Canetto S.S., Erlangsen A., Lapierre S., Lindner R., Draper B., Gallo J.J., Wong C., Wu J. (2016). A systematic review of physical illness, functional disability, and suicidal behaviour among older adults. Aging Ment. Health.

[61] Mitchell J.E., Crow S. (2006). Medical complications of anorexia nervosa and bulimia nervosa. Curr. Opin. Psychiatry.

[62] Salama A. (2009). Drug-induced immune hemolytic anemia. Expert Opin. Drug Saf..

[63] Hesdorffer C.S., Longo D.L. (2015). Drug-Induced Megaloblastic Anemia. N. Engl. J. Med..

[64] Jadoon K.A., Tan G.D., O’Sullivan S.E. (2017). A single dose of cannabidiol reduces blood pressure in healthy volunteers in a randomized crossover study. JCI Insight.

